# Prevalence and characterization of *mecC* MRSA in bovine bulk tank milk in Great Britain, 2017–18

**DOI:** 10.1093/jacamr/dlaa125

**Published:** 2021-02-08

**Authors:** Cheng Cui, Xiaoliang Ba, Mark A Holmes

**Affiliations:** Department of Veterinary Medicine, University of Cambridge, Cambridge, UK

## Abstract

**Objectives:**

To evaluate the current prevalence status of *mecC* MRSA among dairy farms in England and Wales 5 years after a previous survey conducted in 2011–12.

**Methods:**

A convenience sample of 697 dairy farms in England and Wales was used for the study, conducted in 2017–18, testing bulk tank milk samples for the presence of *mecC* MRSA using high salt broth enrichment and chromogenic MRSA agar selection. All putative MRSA isolates were screened by PCR for the presence of *mecA* and *mecC* genes and subjected to antimicrobial susceptibility testing using both the disc diffusion method and VITEK^®^ 2. MRSA isolates were also sequenced for genomic characterization.

**Results:**

*mecC* MRSA were detected on 4 out of 697 dairy farms in England and Wales (prevalence 0.57%, 95% CI 0.16%–1.46%). Three of the *mecC* isolates were ST425 and one was ST4652 (in the CC130 lineage). Two *mecA* MRSA were also isolated: one ST5 and one ST398.

**Conclusions:**

These results indicate that there has been a substantial reduction in the prevalence of *mecC* MRSA in England and Wales with a 72% reduction (2.15% to 0.57%) compared with a previous study. While the levels of *mecA* MRSA remain very low the continued presence of ST398, a livestock-associated MRSA, suggests that this lineage is established in the UK.

## Introduction

MRSA represent a significant healthcare problem causing infections in both people and animals. MRSA typically have high-level resistance to β-lactams. The resistance to β-lactams in staphylococci most commonly results from expression of a *blaZ* gene (encoding a β-lactamase) and/or a *mec* gene (*mecA, mecC or mecB* encoding an alternative PBP2, the target of β-lactams).^1^

The *mecC* gene was initially found among bovine and human isolates from the UK and Denmark in 2011.^2^ Although *mecC* MRSA was first isolated from cattle and humans, it has a wide range of host species, including other livestock species, companion animals and wildlife.^3^

The *mecC* gene has 69% nucleotide identity with *mecA.* As a result, *mecC* MRSA test negative when using molecular detection methods based on the *mecA* gene. For example, agglutination assays for *mecA* encoded PBP2a (using latex beads coated with monoclonal antibodies) will misidentify *mecC* MRSA as being susceptible.^4^ A number of PCR assays^5^ have been developed or adapted to include detection of the *mecC* gene. Phenotypic testing for MRSA based on antimicrobial susceptibility fails to discriminate between *mecA* and *mecC* MRSA and severe and fatal cases of *mecC* MRSA infections have been described in the literature.^6^

Zoonotic transmission of *mecC* MRSA from livestock to humans has been reported,^7^^,^^8^ highlighting the importance of monitoring its prevalence in both human and livestock populations. Paterson *et al*.^9^^,^^10^ reported a 0.45% prevalence rate among 2010 MRSA isolates collected from individual human patients from six microbiology laboratories in England and a 2.15% prevalence rate in bulk tank milk samples collected from 465 dairy farms in England and Wales in 2011–12. In order to provide an updated prevalence of *mecC* MRSA on dairy farms in England and Wales this survey was undertaken using bulk tank milk samples collected in 2017–18.

## Methods

### Bulk tank milk samples and processing

Milk samples (*n *=* *697) from England and Wales were supplied by a commercial milk testing company responsible for more than 95% of quality assurance testing of bulk tank milk from GB dairy farms. The samples were being used for another study between January 2017 and April 2018 and were exploited as a convenience sample for this study. The testing laboratory was asked to provide randomly selected samples although no formal randomization protocol was followed. No farms were sampled more than once, and the date of sampling and the location of each farm were the only metadata available.

The bulk tank milk samples were collected aseptically and stored at 4 °C for up to 5 days before freezing at −20°C prior to testing. The processing of the samples was carried out as described previously.^10^ Briefly, samples were thawed at 37°C and 1 mL of milk was added to 10 mL of 6% NaCl broth (E&O Laboratories, UK). After static incubation at 37°C for 24 h, 50 μL of culture was spread onto MRSA Brilliance 2 plates (Oxoid) and incubated at 37°C for 24 h. Potential MRSA colonies (blue colour) were subcultured on Staph Brilliance 24 plates (Oxoid) and subsequently screened for *mecA*, *mecC* and *femB* by multiplex PCR as described previously.^11^

### Antimicrobial susceptibility testing

Disc diffusion method susceptibility testing for penicillin (Oxoid, 1 unit) and cefoxitin (Oxoid, 30 μg) was performed according to the EUCAST standard (Version 7.1, March 2017). As EUCAST does not define a disc diffusion breakpoint for oxacillin, we also performed oxacillin (Oxoid, 1 μg) disc diffusion according to BSAC (Version 14, January 2015). The VITEK^®^ 2 system was used to validate the antimicrobial susceptibility testing results using an AST-GP79 card with the EUCAST breakpoint parameter set configuration, in accordance with the manufacturer’s instructions.

### Genome sequencing and bioinformatic analysis

Genomic DNA of MRSA isolates was extracted from overnight cultures grown in TSB at 37°C with 200 rpm shaking using the MasterPure Gram-Positive DNA Purification Kit (Cambio, UK). Illumina library preparation, HiSeq sequencing, and genome assembly were carried out as previously described.^7^ The presence of *mecA* or *mecC* genes in all MRSA isolates was confirmed and the multilocus ST was also determined using the sequencing data. The presence of antimicrobial resistance genes in these isolates were determined using the Resfinder gene database to search the assembled genomes using ≥80% identity over ≥60% of the length of the target gene as a threshold for a positive identification. A maximum likelihood core genome phylogenetic tree was produced using an alignment generated by Panaroo^12^ and IQ-TREE 2^13^ to create the tree.

### Ethical review

The study was subject to ethical review at the University of Cambridge, Department of Veterinary Medicine (ref. 2017/012).

### Data availability

The sequencing data for the six isolates described in this paper have been deposited in the European Short Read Archive (ebi.ac.uk) with the following sample accession numbers: QM059, ERS2607810; QM212, ERS2607839; QM312, ERS2607851; QM355, ERS2607859; QM518, ERS2607880; QM652, ERS2607892. A list of accession numbers for other sequence data used in analyses is provided in the Supplementary data (available at *JAC-AMR* Online).

## Results

### Prevalence of mecC MRSA among dairy farm bulk milk samples

**Figure 1.**
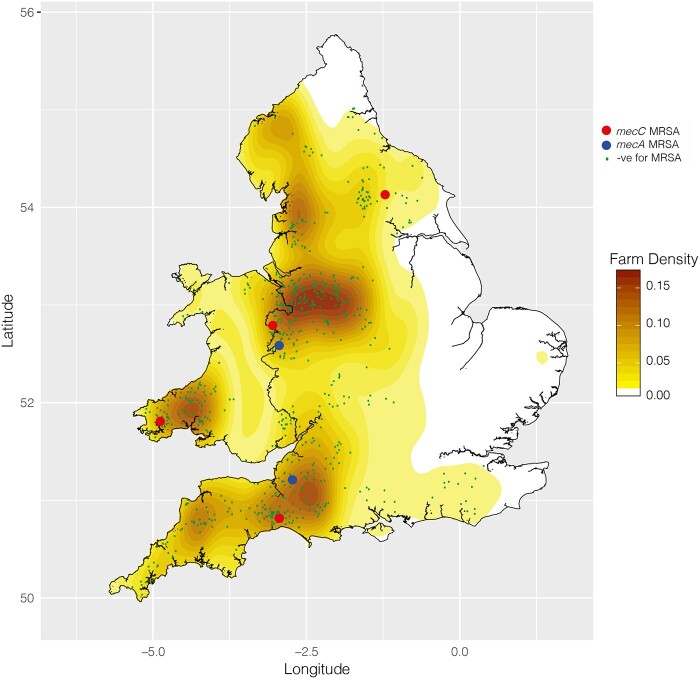
Geographical distribution of farms included in the survey with results. Map of England and Wales showing the location of farms tested and their results. Farms testing negative are shown as green dots, *mecC* MRSA-positive farms are shown with larger red circles and *mecA* MRSA-positive farms are shown with larger blue circles. A heatmap showing the density of dairy farms per square kilometre is also displayed on the map.

Four dairy farms from a total of 697 were positive for *mecC* MRSA, which represents a farm level prevalence rate of 0.57% (95% CI 0.16%–1.46%) in England and Wales. Three of the positive farms were in England and one was in Wales. Figure 1 maps the locations of farms sampled, the locations of the positive farms, and a heat map of the density of dairy farms across England and Wales. A comparison of the distribution of the sampled farms with the average density of dairy farms shows that the sampling distribution matches that of the farms being sampled with no obvious deviant clustering.

### Characterization of bovine mecC MRSA

All of the four *mecC* MRSA isolates were resistant to penicillin, oxacillin and cefoxitin according to disc diffusion antimicrobial susceptibility testing (Table 1). Further antimicrobial susceptibility testing on these isolates using the VITEK^®^ 2 system confirmed the resistance to these three antimicrobials and also reported that all isolates were resistant to cefalotin, a first-generation cephalosporin antibiotic. All *mecC* isolates had *blaZ* and *mecC* genes identical to those described in the original *mecC* paper,^2^ but no other antimicrobial resistance genes.

**Table 1.** Phenotypic and genotypic characteristics of MRSA isolates in this study

Isolate	County	*mec* gene	ST	spa type	Oxacillin		Cefoxitin		Penicillin		Cefalotin	Human immune evasion cluster		
					IZD (mm)	VITEK	IZD (mm)	VITEK	IZD (mm)	VITEK	VITEK	*chp*	*scn*	*sak*
QM059	North Yorkshire	*mecC*	4652	t843	7	R	18	R	15	R	R	−	−	−
QM212	Somerset	*mecA*	5	t002	6	R	10	R	6	R	R	+	+	+
QM312	Shropshire	*mecA*	398	t034	6	R	15	R	6	R	R	+	+	+
QM355	Shropshire	*mecC*	425	t10855	11	R	20	R	14	R	R	−	−	−
QM518	Devon	*mecC*	425	t6292	6	R	18	R	10	R	R	−	−	−
QM652	Pembrokeshire	*mecC*	425	t6292	6	R	17	R	10	R	R	−	−	−

IZD, inhibition zone diameter; R, resistant.

6 mm in disc diffusion indicates no inhibition zone.

For penicillin, resistant breakpoint for disc diffusion is <26 mm diameter.

For cefoxitin, resistant breakpoint for disc diffusion is <22 mm diameter.

For oxacillin, resistant breakpoint for disc diffusion is ≤14 mm diameter (BSAC).

spa types were determined from the sequencing data using spaTyper 1.0 (https://cge.cbs.dtu.dk/services/spaTyper-1.0/).

**Figure 2.**
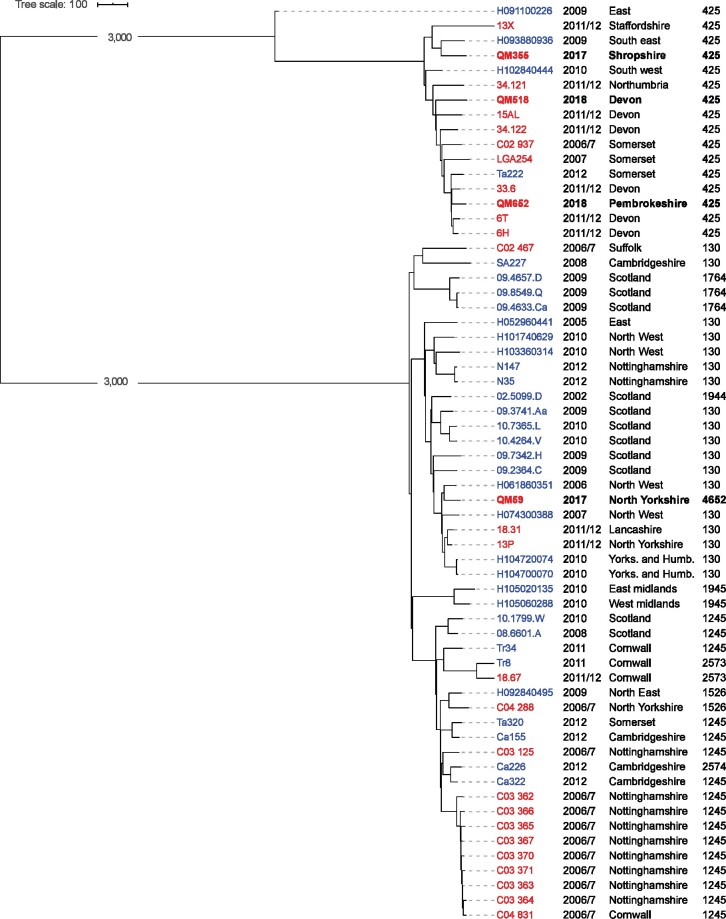
Core-genome phylogenetic tree showing the *mecC* MRSA isolates found in this study (indicated by bold text) together with other published *mecC* isolates from the UK.^2^^,^^10^^,^^16^ The core genome alignment was produced using Panaroo^12^ and the maximum likelihood tree generated using IQ-TREE 2.^13^ The year of isolation, geographical region MLSTs are provided in the text next to the isolate names. Isolates with names in blue text came from human samples. Isolates with names in red text were of bovine origin. The branches of the trees separating the two different lineages (CC425 and CC130) are not shown to scale in order to make the relationships within the two clades easier to see. Accession numbers for sequencing data for all isolates in the tree are provided in the Supplementary data.

A phylogenetic tree showing the four positive isolates in the context of previous *mecC* isolates found in the UK is shown in Figure 2. Three of the isolates were ST425 and one was ST4625 (a single locus variant of ST130) representing the two main lineages found in the UK.

### Prevalence and characteristics of mecA MRSA from GB bulk milk

Two *mecA* MRSA were also identified in the survey giving a prevalence of 0.29% (95% CI 0.08%–1.04%). The location of the positive farms is shown in Figure 1. Both isolates were resistant to penicillin, oxacillin and cefoxitin based on the results of disc diffusion and VITEK^®^ 2 (Table 1). The VITEK^®^ 2 results also showed that both isolates were resistant to cefalotin, erythromycin and clindamycin and isolate QM312 was also resistant to ceftiofur and cefquinome. Isolate QM212 belongs to ST5 and possessed *mecA*, *blaZ* and *ermA* antimicrobial resistance genes. Isolate QM312 was an ST398 MRSA associated with livestock-associated MRSA epidemiology. This isolate possessed all three canonical SNPs associated with the livestock-independent clade of this lineage and, along with the ST5 MRSA, possessed the immune evasion cluster genes *scn*, *chp* and *sak.*^14^ QM312 possessed an *ermC* gene in addition to *mecA*.

## Discussion

In this study, we conducted a follow-up prevalence study of *mecC* MRSA on dairy farms in England and Wales 5 years after the previous survey.^10^ We were unable to include Scotland in this survey although in the previous study no MRSA were found in Scottish dairy herds. Four out of 697 dairy farms were found to have *mecC* MRSA, which gives an estimated prevalence of 0.57%, compared with a 2.15% prevalence reported for 2011–12, a reduction of 72%.^10^ The original *mecC* prevalence study employed formal methodology to ensure random selection of dairy farms whereas this study used a convenience sample. Nonetheless an examination of the distribution of farms sampled does not indicate any obvious bias in this sample. Furthermore, the results from this study are similar to another study, published recently, which sampled 363 farms in 2015–16 and reported two *mecC* MRSA positives (0.5%).^15^

Although the frequency of *mecC* MRSA remains low, its presence in dairy cattle poses a zoonotic risk to humans. Two case studies have demonstrated the transmission of *mecC* MRSA from livestock to people evidenced using molecular epidemiology.^7^^,^^8^ Three human prevalence surveys have been performed in the UK. A study performed in 2011–12 reported that *mecC* was responsible for 0.45% of MRSA^9^ in six hospitals across the UK and a 2012–13 study in the East of England reported a *mecC* MRSA rate of 0.52%.^16^ A more recent study of human MRSA isolates from North-West England, collected in 2015, reported a *mecC* MRSA prevalence of 0.07%,^17^ a reduction in the human prevalence that appears to parallel the fall seen in livestock. Any reduction in livestock of this MRSA is to be welcomed and will reduce the potential for zoonotic transmission.

We can only speculate on what may have caused a reduction in prevalence of *mecC* MRSA in UK dairy farms. The samples in this prevalence study were collected in 2017–18, which coincides with the end of the ‘UK five year AMR strategy 2013 to 2018’ published by the UK Department of Health^18^ and it seems reasonable to attribute the observed decrease to the successful implementation of this strategy, although many other events or phenomena may be responsible. According to the UK One Health Report published in early 2019,^19^ an overall reduction of 19% in antibiotic usage was achieved in people and animals between 2013 and 2017 with a drop of 35% in animals (436 to 282 tonnes). Antibiotic use is an obvious driver of antibiotic resistance and the use of β-lactams, particularly cephalosporins, on dairy farms is likely to select for *mecC* MRSA due to the differential affinity of the *mecC* PBP2a for cephalosporins.^20^ The 2018 UK Veterinary Antibiotic Resistance and Sales Surveillance Report^21^ reports a 50% reduction in third/fourth-generation cephalosporin use on dairy farms from 2017 to 2018.

Apart from *mecC* MRSA, this study has also identified two *mecA* MRSA-positive farms in England, at similar levels to those seen in 2011–12. One *mecA* MRSA isolate belongs to ST5, a typical human *S. aureus* lineage and the other to the livestock-associated MRSA lineage ST398. As the ST398 is a member of the livestock independent lineage, with the human immune evasion cluster, it is possible that both these *mecA* MRSA were of recent human origin. These findings are notable but not alarming at this time.

### Conclusions

This study has found a welcome decrease in *mecC* MRSA prevalence on the dairy farms in England in comparison to that of 2011–12 that may be the result of improved antibiotic stewardship in the UK. In light of the zoonotic potential of this MRSA from livestock to humans, it is important to continue to monitor levels of *mecC* MRSA in agriculture and the environment.

## Funding

The research was funded by UK Medical Research Council awards MR/P007201/1 and MR/S013660/1. Cheng Cui was supported by a British Council fellowship.

## Transparency declarations

None to declare.

## Supplementary data

A list of accession numbers is available as Supplementary data at *JAC-AMR* Online.

## Supplementary Material

dlaa125_Supplementary_Data
